# Representations for the Generalized Drazin Inverse of the Sum in a Banach Algebra and Its Application for Some Operator Matrices

**DOI:** 10.1155/2015/156934

**Published:** 2015-02-01

**Authors:** Xiaoji Liu, Xiaolan Qin

**Affiliations:** Faculty of Science, Guangxi University for Nationalities, Nanning 530006, China

## Abstract

We investigate additive properties of the generalized Drazin inverse in a Banach algebra *A*. We find explicit expressions for the generalized Drazin inverse of the sum *a* + *b*, under new conditions on *a*, *b* ∈ *A*. As an application we give some new representations for the generalized Drazin inverse of an operator matrix.

## 1. Introduction

Let *A* be a complex Banach algebra with unite 1. We use *σ*(*a*) to denote the spectrum of *a* ∈ *A*. The sets of all nilpotent and quasinilpotent elements (*σ*(*a*) = {0}) of *A* will be denoted by *A* ^nil^ and *A* ^qnil^, respectively.

The generalized Drazin inverse of *a* ∈ *A* (introduced by Koliha in [1]) is the element *b* ∈ *A* which satisfies

(1) $$\begin{array}{c} xax=x,\;\;ax=xa,\;\;a-a^{2}x\in A^{\text{qnil}}. \end{array}$$

If there exists the generalized Drazin inverse, then the generalized Drazin inverse of *a* is unique and is denoted by *a* ^*d*^. The set of all generalized Drazin invertible elements of *A* is denoted by *A* ^*d*^. For interesting properties of the generalized Drazin inverse see [2–6]. For a complete treatment of the generalized Drazin inverse, see [7, Chapter 2].

If *p* = *p* ^2^ ∈ *A* is an idempotent, we denote $\bar{p}=1-p$. We can represent element *a* ∈ *A* as

(2) $$\begin{array}{c} a={\left[\begin{array}{cc} a_{11} & a_{12}\\ a_{21} & a_{22} \end{array}\right]}_{p}, \end{array}$$

where *a*_11_ = *pap*, $a_{12}=pa\bar{p}$, $a_{21}=\bar{p}ap$, and $a_{22}=\bar{p}a\bar{p}$.

Let *a* ∈ *A* ^*d*^ and *a* ^*π*^ = 1 − *aa* ^*d*^ be the spectral idempotent of *a* corresponding to {0}. It is well known that *a* ∈ *A* can be represented in the following matrix form ([7, Chapter 2]):

(3) $$\begin{array}{c} a={\left[\begin{array}{cc} a_{1} & 0\\ 0 & a_{2} \end{array}\right]}_{p}, \end{array}$$

relative to *p* = *aa* ^*d*^, where *a*_1_ is invertible in the algebra *pAp*, *a* ^*d*^ is its inverse in *pAp*, and *a*_2_ is quasinilpotent in the algebra $\bar{p}A\bar{p}$. Thus, the generalized Drazin inverse of *a* can be expressed as

(4) $$\begin{array}{c} a^{d}={\left[\begin{array}{cc} a_{1}^{d} & 0\\ 0 & 0 \end{array}\right]}_{p}. \end{array}$$

Obviously, if *a* ∈ *A* ^qnil^, then *a* is generalized Drazin invertible and *a* ^*d*^ = 0.

In this paper, we first give the formulas of (*a* + *b*)^*d*^ under the conditions *ab* = *bab* ^*π*^ and *ab* = *a* ^*π*^ *bab* ^*π*^, respectively. Then we will apply these formulas to provide some representations for the generalized Drazin inverse of the operator matrix $M=\left[\begin{array}{cc} A & B\\ C & D \end{array}\right]$ under some conditions.

## 2. Main Results

First we start the following result which is proved in [8] for matrices, extended in [9] for a bounded linear operator and in [10] for arbitrary elements in a Banach algebra.

**Lemma 1 (see [10, Theorem 2.3]).**

Let *x*, *y* ∈ *A* and *p* ∈ *A* be an idempotent. Assume that *x* and *y* are represented as

(5) $$\begin{array}{c} x={\left[\begin{array}{cc} a & 0\\ c & b \end{array}\right]}_{p},\;\;y={\left[\begin{array}{cc} b & c\\ 0 & a \end{array}\right]}_{p}. \end{array}$$

(i) If *a* ∈ (*pAp*)^*d*^ and $b\in {\left(\bar{p}A\bar{p}\right)}^{d}$, then *x* and *y* are generalized Drazin invertible, and
  (6) $$\begin{array}{c} x^{d}={\left[\begin{array}{cc} a^{d} & 0\\ u & b^{d} \end{array}\right]}_{p},\;\;y^{d}={\left[\begin{array}{cc} b^{d} & u\\ 0 & a^{d} \end{array}\right]}_{p}, \end{array}$$
  where
  (7) $$\begin{array}{c} u=\overset{\infty }{\underset{n=0}{\sum }}{\left(b^{d}\right)}^{n+2}ca^{n}a^{\pi }+\overset{\infty }{\underset{n=0}{\sum }}b^{\pi }b^{n}c{\left(a^{d}\right)}^{n+2}-b^{d}ca^{d}. \end{array}$$
(ii) If *x* ∈ *A* ^*d*^ and *a* ∈ (*pAp*)^*d*^, then $b\in {\left(\bar{p}A\bar{p}\right)}^{d}$ and *x* ^*d*^ and *y* ^*d*^ are given by (6) and (7).

**Lemma 2 (see [11, Lemma 2.1]).**

Let *a*, *b* ∈ *A* ^*qnil*^. If *ab* = *ba* or *ab* = 0, then *a* + *b* ∈ *A* ^*qnil*^.

The following result is a generalization of [10, Corollary 3.4].

**Theorem 3 .**

If *a* ∈ *A* ^*qnil*^, *b* ∈ *A* ^*d*^, and *ab* = *bab* ^*π*^, then *a* + *b* ∈ *A* ^*d*^ and

(8) $$\begin{array}{c} {\left(a+b\right)}^{d}=b^{d}+\overset{\infty }{\underset{n=0}{\sum }}{\left(b^{d}\right)}^{n+2}a{\left(a+b\right)}^{n}. \end{array}$$

**Proof**

First, suppose that *b* ∈ *A* ^qnil^. Therefore, *b* ^*π*^ = 1 and from *ab* = *bab* ^*π*^ we obtain *ab* = *ba*. Using Lemma 2, *a* + *b* ∈ *A* ^qnil^ and (8) holds.

Now we assume *b* is not quasinilpotent, using matrix representations of *a* and *b* relative to *p* = *bb* ^*d*^. We have

(9) $$\begin{array}{c} b={\left[\begin{array}{cc} b_{1} & 0\\ 0 & b_{2} \end{array}\right]}_{p},\;\;b^{d}={\left[\begin{array}{cc} b_{1}^{d} & 0\\ 0 & 0 \end{array}\right]}_{p}, \end{array}$$

where *b*_1_ ∈ (*pAp*)^−1^, $b_{2}\in {\left(\bar{p}A\bar{p}\right)}^{\text{qnil}}$.

Let us represent

(10) $$\begin{array}{c} a={\left[\begin{array}{cc} a_{1} & a_{2}\\ a_{3} & a_{4} \end{array}\right]}_{p}. \end{array}$$

From *ab* = *bab* ^*π*^ and

(11) $$\begin{array}{c} ab={\left[\begin{array}{cc} a_{1}b_{1} & a_{2}b_{2}\\ a_{3}b_{1} & a_{4}b_{2} \end{array}\right]}_{p},\;\;bab^{\pi }={\left[\begin{array}{cc} 0 & b_{1}a_{2}\\ 0 & b_{2}a_{4} \end{array}\right]}_{p}, \end{array}$$

we obtain *a*_1_ *b*_1_ = 0 and *a*_3_ *b*_1_ = 0. Since *b*_1_ is invertible, we have *a*_1_ = 0 and *a*_3_ = 0.

Hence we have

(12) $$\begin{array}{c} a+b={\left[\begin{array}{cc} b_{1} & a_{2}\\ 0 & a_{4}+b_{2} \end{array}\right]}_{p}. \end{array}$$

The condition *ab* = *bab* ^*π*^ implies that *a*_4_ *b*_2_ = *b*_2_ *a*_4_. Hence, using Lemma 2, we get *a*_4_ + *b*_2_ ∈ *A* ^qnil^. By Lemma 1, we obtain that *a* + *b* ∈ *A* ^*d*^ and

(13) $$\begin{array}{c} {\left(a+b\right)}^{d}={\left[\begin{array}{cc} b_{1}^{d} & u\\ 0 & 0 \end{array}\right]}_{p}, \end{array}$$

where

(14) $$\begin{array}{c} u=\overset{\infty }{\underset{n=0}{\sum }}{\left(b_{1}^{d}\right)}^{n+2}a_{2}{\left(a_{4}+b_{2}\right)}^{n}. \end{array}$$

Now from (14), using the matrix representation of *b* ^*d*^, *a*, and *a* + *b*, we easily obtain formula (8) of the theorem.

The next result is a generalization of [12, Theorem 2.2] and [10, Example 4.5].

**Theorem 4 .**

Let *a*, *b* ∈ *A* ^*d*^. If *ab* = *a* ^*π*^ *bab* ^*π*^, then *a* + *b* ∈ *A* ^*d*^ and

(15) a+bd=bπad+bdaπ+∑n=0∞bdn+2aa+bnaπ+bπ∑n=0∞a+bnbadn+2−∑n=0∞ ∑k=0∞bdk+1aa+bn+kbadn+2−∑n=0∞bdn+2aa+bnbad.

**Proof**

If *a* is quasinilpotent, we can apply Theorem 3 and we obtain (15) for this particular case. Now we assume that *a* is neither invertible nor quasinilpotent and consider the following matrix representations of *a*, *a* ^*d*^, and *b* relative to the *p* = *aa* ^*d*^:

(16) $$\begin{array}{c} a={\left[\begin{array}{cc} a_{1} & 0\\ 0 & a_{2} \end{array}\right]}_{p},\;\;a^{d}={\left[\begin{array}{cc} a_{1}^{d} & 0\\ 0 & 0 \end{array}\right]}_{p},\;\;b={\left[\begin{array}{cc} b_{1} & b_{2}\\ b_{3} & b_{4} \end{array}\right]}_{p}. \end{array}$$

The condition *ab* = *a* ^*π*^ *bab* ^*π*^ implies that *a*_1_ *b*_1_ = 0 and *a*_1_ *b*_2_ = 0. Since *a*_1_ is invertible, we have *b*_1_ = 0 and *b*_2_ = 0.

Thus, *b* can be represented as

(17) $$\begin{array}{c} b={\left[\begin{array}{cc} 0 & 0\\ b_{3} & b_{4} \end{array}\right]}_{p}. \end{array}$$

Therefore, $b_{4}\in {\left(\bar{p}A\bar{p}\right)}^{d}$ and, from Lemma 1, we have

(18) $$\begin{array}{c} b^{d}={\left[\begin{array}{cc} 0 & 0\\ {\left(b_{4}^{d}\right)}^{2}b_{3} & b_{4}^{d} \end{array}\right]}_{p},\;\;b^{\pi }={\left[\begin{array}{cc} p & 0\\ -b_{4}^{d}b_{3} & b_{4}^{\pi } \end{array}\right]}_{p}. \end{array}$$

From *ab* = *a* ^*π*^ *bab* ^*π*^ and

(19) $$\begin{array}{c} ab={\left[\begin{array}{cc} 0 & 0\\ a_{2}b_{3} & a_{2}b_{4} \end{array}\right]}_{p},\\ a^{\pi }bab^{\pi }={\left[\begin{array}{cc} 0 & 0\\ b_{3}a_{1}-b_{4}a_{2}b_{4}^{d}b_{3} & b_{4}a_{2}b_{4}^{\pi } \end{array}\right]}_{p}, \end{array}$$

we obtained *a*_2_ *b*_4_ = *b*_4_ *a*_2_ *b*_4_^*π*^. From Theorem 3, we get *a*_2_ + *b*_4_ ∈ *A* ^*d*^ and

(20) $$\begin{array}{c} {\left(a_{2}+b_{4}\right)}^{d}=b_{4}^{d}+\overset{\infty }{\underset{n=0}{\sum }}{\left(b_{4}^{d}\right)}^{n+2}a_{2}{\left(a_{2}+b_{4}\right)}^{n}. \end{array}$$

Further, applying Lemma 1 to *a* + *b*, we get

(21) $$\begin{array}{c} {\left(a+b\right)}^{d}=\left[\begin{array}{cc} a_{1}^{d} & 0\\ u & {\left(a_{2}+b_{4}\right)}^{d} \end{array}\right], \end{array}$$

where

(22) u=∑n=0∞a2+b4dn+2b3a1na1π +∑n=0∞a2+b4πa2+b4nb3a1dn+2 −a2+b4db3a1d.

Observe that since *a*_1_ ∈ (*pAp*)^−1^, then *a*_1_^*π*^ = 0.

Hence, the expression of *u* reduces to

(23) u=∑n=0∞a2+b4πa2+b4nb3a1dn+2−a2+b4db3a1d.

From *a*_2_ *b*_4_ = *b*_4_ *a*_2_ *b*_4_^*π*^ we get = *a*_2_ *b*_4_(*b*_4_^*d*^)^2^ = *b*_4_ *a*_2_ *b*_4_^*π*^(*b*_4_^*d*^)^2^ = 0.

Hence, from formula (20) and *a*_2_ *b*_4_^*d*^ = 0, we have

(24) a2+b4π=p¯−a2+b4·b4d+∑n=0∞b4dn+2a2a2+b4n=p¯−b4b4d+∑n=0∞b4dn+2a2a2+b4n=b4π−∑n=0∞b4dn+1a2a2+b4n.

Then substituting (20) and (24) in (22), we get

(25) u=∑n=0∞b4πa2+b4nb3a1dn+2 −∑n=0∞ ∑k=0∞b4dk+1a2a2+b4n+kb3a1dn+2 −b4db3a1d−∑n=0∞b4dn+2a2a2+b4nb3a1d.

Now, replacing *u* by the above expression and considering matrix representations of *a* and *b*, after direct computations, we obtain the formula (15) for (*a* + *b*)^*d*^.

## 3. Applications

In this section, we give some formulas for the generalized Drazin inverse of a 2 × 2 operator matrix under some conditions.

Finding an explicit representation for the generalized Drazin inverse of an operator matrix $M=\left[\begin{array}{cc} A & B\\ C & D \end{array}\right]$ in terms of *A*, *B*, *C*, *D* and related generalized Drazin inverse has been studied by several authors [9, 13–15]. Djordjević and Stanimirović [9] generalize the well-known result in [8, 16] concerning the Drazin inverse of block 2 × 2 upper triangular matrices to the generalized Drazin inverse for a block triangular operator matrix. Further, they consider the case where *BC* = 0, *BD* = 0, and *DC* = 0.

This section is devoted to the generalized Drazin inverse of 2 × 2 operator matrix:

(26) $$\begin{array}{c} M=\left[\begin{array}{cc} A & B\\ C & D \end{array}\right], \end{array}$$

where *A* ∈ **B**(*X*) and *D* ∈ **B**(*Y*) are generalized Drazin invertible.

Next we will state some auxiliary lemmas.

**Lemma 5 (see [2, 3]).**

Let *A* and *D* be generalized Drazin invertible and let *M* be matrix of form (26). If *BC* = 0 and *BD* = 0, then

(27) $$\begin{array}{c} M^{d}=\left[\begin{array}{cc} A^{d} & {\left(A^{d}\right)}^{2}B\\ X_{0} & D^{d}+X_{1}B \end{array}\right], \end{array}$$

where

(28) Xn=∑i=0∞Ddi+n+2CAiAπ +Dπ∑i=0∞DiCAdi+n+2 −∑i=0nDdi+1CAdn−i+1, n≥0.

**Lemma 6 (see [17, Lemma 3.1]).**

If *M* is matrix of form (26), such that *A* is generalized Drazin invertible, *D* is quasinilpotent, and *BD* ^*n*^ *C* = 0 for any nonnegative integer *n*, then *M* is generalized Drazin invertible and

(29) $$\begin{array}{c} M^{d}=\left[\begin{array}{cc} A^{d} & \Gamma \\ \Delta & \Delta A\Gamma \end{array}\right], \end{array}$$

where

(30) $$\begin{array}{c} \Gamma =\overset{\infty }{\underset{n=0}{\sum }}{\left(A^{d}\right)}^{n+2}BD^{n},\;\;\Delta =\overset{\infty }{\underset{n=0}{\sum }}D^{n}C{\left(A^{d}\right)}^{n+2}. \end{array}$$

**Lemma 7 .**

Let *A* ∈ *C* ^*n*×*n*^. Then (*AA* ^*π*^)^*d*^ = 0, (*A* ^2^ *A* ^*d*^)^*d*^ = *A* ^*d*^, (*A* ^2^ *A* ^*d*^)^*π*^ = *A* ^*π*^, and Ind(*AA* ^*π*^) = Ind(*A*) and Ind(*A* ^2^ *A* ^*d*^) = 1.

**Proof**

The Jordan canonical form of *X* permits us to write *A* = *S*(*C* ⊕ *N*)*S* ^−1^, where *S* and *C* are nonsingular, and *N* is nilpotent with index Ind(*A*). Thus *A*_*d*_ = *S*(*C* ^−1^ ⊕ 0)*S* ^−1^. Now, it is evident that *A* ^2^ *A* ^*d*^ = *S*(*C* ⊕ 0)*S* ^−1^ and *AA* ^*π*^ = *S*(0 ⊕ *N*)*S* ^−1^, which lead to the affirmations of this lemma.

In [9, Theorem 5.3] authors gave an explicit representation for *M* ^*d*^ under conditions *BC* = 0, *DC* = 0, and *BD* = 0. Here we replace the last two conditions by the two weaker conditions *DC* = *D* ^*π*^ *CAA* ^*π*^ and *BD* = *AA* ^*π*^ *B*.

**Theorem 8 .**

Let *A* and *D* be generalized Drazin invertible and let *M* be matrix of form (26). If *AA* ^*π*^ *B* = *BD*, *DC* = *D* ^*π*^ *CAA* ^*π*^ and *BC* = 0. Then

(31) $$\begin{array}{c} M^{d}=\left[\begin{array}{cc} A^{d} & {\left(A^{d}\right)}^{2}B+\overset{\infty }{\underset{n=0}{\sum }}A^{n}B{\left(D^{d}\right)}^{n+2}\\ C{\left(A^{d}\right)}^{2} & D^{d}+C{\left(A^{d}\right)}^{3}B+\overset{\infty }{\underset{n=1}{\sum }}\;\overset{n}{\underset{i=1}{\sum }}D^{i-1}CA^{n-i}B{\left(D^{d}\right)}^{n+2}‍ \end{array}\right]. \end{array}$$

**Proof**

We can split matrix *M* as *M* = *P* + *Q*, where

(32) $$\begin{array}{c} P=\left[\begin{array}{cc} AA^{\pi } & 0\\ 0 & D \end{array}\right],\;\;Q=\left[\begin{array}{cc} A^{2}A^{d} & B\\ C & 0 \end{array}\right],\\ P^{d}=\left[\begin{array}{cc} 0 & 0\\ 0 & D^{d} \end{array}\right],\;\;P^{\pi }=\left[\begin{array}{cc} I & 0\\ 0 & D^{\pi } \end{array}\right]. \end{array}$$

Since *DC* = *D* ^*π*^ *CAA* ^*π*^ and *AA* ^*π*^ *B* = *BD*, we have

(33) $$\begin{array}{c} D^{d}C={\left(D^{d}\right)}^{2}DC={\left(D^{d}\right)}^{2}D^{\pi }CAA^{\pi }=0,\\ DCA^{d}=D^{\pi }CAA^{\pi }A^{d}=0,\\ A^{d}BD=A^{d}AA^{\pi }B=0. \end{array}$$

From *BC* = 0 and applying Lemma 5 to *Q*, we obtain

(34) $$\begin{array}{c} {\left(Q^{d}\right)}^{i}=\left[\begin{array}{cc} {\left(A^{d}\right)}^{i} & {\left(A^{d}\right)}^{i+1}B\\ X_{i-1} & X_{i}B \end{array}\right],\\ Q^{\pi }=\left[\begin{array}{cc} A^{\pi } & -A^{d}B\\ -CA^{d} & I-C{\left(A^{d}\right)}^{2}B \end{array}\right], \end{array}$$

where *X*_*n*_ is defined in (28). From *D* ^*d*^ *C* = 0 and *DCA* ^*d*^ = 0, we get *X*_*n*_ = *C*(*A* ^*d*^)^*n*+2^.

Since *AA* ^*π*^ *B* = *BD* and *DC* = *D* ^*π*^ *CAA* ^*π*^, we obtain *PQ* = *P* ^*π*^ *QPQ* ^*π*^. Applying Theorem 4, we get

(35) P+Qd=QπPd+QdPπ +∑n=0∞Qdn+2PP+QnPπ +Qπ∑n=0∞P+QnQPdn+2 −∑n=0∞ ∑k=0∞Qdk+1PP+Qn+kQPdn+2 −∑n=0∞Qdn+2PP+QnQPd.

From *A* ^*d*^ *BD* = 0, we have

(36) $$\begin{array}{c} Q^{\pi }P^{d}=P^{d},\;\;Q^{d}P^{\pi }=Q^{d},\;\;Q^{d}P=0. \end{array}$$

Hence from (35), we obtain

(37) $$\begin{array}{c} {\left(P+Q\right)}^{d}=P^{d}+Q^{d}+Q^{\pi }\overset{\infty }{\underset{n=0}{\sum }}{\left(P+Q\right)}^{n}Q{\left(P^{d}\right)}^{n+2}. \end{array}$$

Since *A* ^*d*^ *BD* = 0, we have

(38) $$\begin{array}{c} Q^{\pi }Q{\left(P^{d}\right)}^{2}=B{\left(D^{d}\right)}^{2}. \end{array}$$

The conditions *BC* = 0 and *BD* = *AA* ^*π*^ *B*  imply that *BD* ^*n*^ *C* = 0. From *BD* ^*n*^ *C* = 0 and *A* ^*d*^ *BD* = 0, we get

(39) Qπ∑n=1∞P+QnQPdn+2  =0∑n=1∞AnBDdn+20∑n=1∞ ∑i=1nDi−1CAn−iBDdn+2‍.

From (36), (38), and (39) it follows (31).

The proof is finished.

Since

(40) $$\begin{array}{c} \left[\begin{array}{cc} A & B\\ C & D \end{array}\right]=\left[\begin{array}{cc} 0 & I_{n}\\ I_{m} & 0 \end{array}\right]\left[\begin{array}{cc} D & C\\ B & A \end{array}\right]\left[\begin{array}{cc} 0 & I_{m}\\ I_{n} & 0 \end{array}\right], \end{array}$$

we can obtain the following result, applying Theorem 8 to $\left[\begin{array}{cc} D & C\\ B & A \end{array}\right]$.

**Theorem 9 .**

Let *A* and *D* be generalized Drazin invertible and let *M* be matrix of form (26). If *DD* ^*π*^ *C* = *CA*, *AB* = *A* ^*π*^ *BDD* ^*π*^ and *CB* = 0. Then

(41) $$\begin{array}{c} M^{d}=\left[\begin{array}{cc} A^{d}+B{\left(D^{d}\right)}^{3}C+\overset{\infty }{\underset{n=1}{\sum }}\;\overset{n}{\underset{i=1}{\sum }}A^{n-i}BD^{i-1}C{\left(A^{d}\right)}^{n+2} & B{\left(D^{d}\right)}^{2}\\ {\left(D^{d}\right)}^{2}C+\overset{\infty }{\underset{n=0}{\sum }}D^{n}C{\left(A^{d}\right)}^{n+2} & D^{d} \end{array}\right]. \end{array}$$

**Theorem 10 .**

Let *A*, *D*, and *BC* be generalized Drazin invertible and let *M* be matrix of form (26). If *AB* = *A* ^*π*^ *BD*, *DC* = *D* ^*π*^ *CA* and *BC* = 0. Then

(42) $$\begin{array}{c} M^{d}=\left[\begin{array}{cc} A^{d} & \overset{\infty }{\underset{n=0}{\sum }}A^{n}B{\left(D^{d}\right)}^{n+2}\\ \overset{\infty }{\underset{n=0}{\sum }}D^{n}C{\left(A^{d}\right)}^{n+2} & D^{d}+\overset{\infty }{\underset{n=1}{\sum }}\;\overset{n-1}{\underset{i=0}{\sum }}D^{i}CA^{n-i-1}B{\left(D^{d}\right)}^{n+2}‍ \end{array}\right]. \end{array}$$

**Proof**

We can split matrix *M* as *M* = *P* + *Q*, where

(43) $$\begin{array}{c} P=\left[\begin{array}{cc} A & 0\\ 0 & D \end{array}\right],\;\;Q=\left[\begin{array}{cc} 0 & B\\ C & 0 \end{array}\right], \end{array}$$

(44) $$\begin{array}{c} P^{d}=\left[\begin{array}{cc} A^{d} & 0\\ 0 & D^{d} \end{array}\right],\;\;P^{\pi }=\left[\begin{array}{cc} A^{\pi } & 0\\ 0 & D^{\pi } \end{array}\right]. \end{array}$$

Since

(45) $$\begin{array}{c} Q^{2}=\left[\begin{array}{cc} BC & 0\\ 0 & CB \end{array}\right],\;\;Q^{3}=\left[\begin{array}{cc} 0 & BCB\\ CBC & 0 \end{array}\right], \end{array}$$

from *BC* = 0, it is easy to get *Q* ^3^ = 0. Since *Q* is nilpotent, we have *Q* ^*d*^ = 0. Applying Theorem 4 to the particular case, we get

(46) $$\begin{array}{c} {\left(P+Q\right)}^{d}=P^{d}+\overset{\infty }{\underset{n=0}{\sum }}{\left(P+Q\right)}^{n}Q{\left(P^{d}\right)}^{n+2}. \end{array}$$

The conditions *AB* = *A* ^*π*^ *BD* and *BC* = 0  imply that *BD* ^*n*^ *C* = 0, for *n* ≥ 0, so we get

(47) ∑n=0∞P+QnQPdn+2 =0∑n=0∞AnBDdn+2∑n=0∞DnCAdn+2∑n=1∞ ∑i=0n−1DiCAn−i−1BDdn+2‍.

From (44) and (47) it follows (42).

The proof is finished.

**Theorem 11 .**

Let *A* and *D* be generalized Drazin invertible and let *M* be matrix of form (26). If *AA* ^*π*^ *B* = *BD* ^2^ *D* ^*d*^, *D* ^2^ *D* ^*d*^ *C* = *D* ^*π*^ *CAA* ^*π*^ and *BD* ^*n*^ *C* = 0 for any nonnegative integer *n*. Then

(48) $$\begin{array}{c} M^{d}=\left[\begin{array}{cc} A^{d} & \Gamma +\overset{\infty }{\underset{n=0}{\sum }}A^{n}B{\left(D^{d}\right)}^{n+2}\\ \Delta & D^{d}+\Delta A\Gamma +\overset{\infty }{\underset{n=1}{\sum }}\;\overset{n-1}{\underset{i=0}{\sum }}D^{i}CA^{n-i-1}B{\left(D^{d}\right)}^{n+2}‍ \end{array}\right], \end{array}$$

where Γ and Δ are defined in (30).

**Proof**

We can split matrix *M* as *M* = *P* + *Q*, where

(49) $$\begin{array}{c} P=\left[\begin{array}{cc} AA^{\pi } & 0\\ 0 & D^{2}D^{d} \end{array}\right],\;\;Q=\left[\begin{array}{cc} A^{2}A^{d} & B\\ C & DD^{\pi } \end{array}\right],\\ P^{d}=\left[\begin{array}{cc} 0 & 0\\ 0 & D^{d} \end{array}\right],\;\;P^{\pi }=\left[\begin{array}{cc} I & 0\\ 0 & D^{\pi } \end{array}\right]. \end{array}$$

From *AA* ^*π*^ *B* = *BD* ^2^ *D* ^*d*^ and *D* ^2^ *D* ^*d*^ *C* = *D* ^*π*^ *CAA* ^*π*^, we have

(50) DdC=Dd3D2C=Dd2D2DdC=Dd2DπCAAπ=0,

(51) AdBDd=AdBD2Dd3=AdBD2DdDd2=AdAAπBDd2=0,

so we get *D* ^*π*^ *C* = *C*.

Note that *DD* ^*π*^ is quasinilpotent, *D* ^*π*^ *C* = *C*, and *B*(*DD* ^*π*^)^*n*^ *C* = *BD* ^*n*^ *D* ^*π*^ *C* = *BD* ^*n*^ *C* = 0 for any nonnegative integer *n*; we can apply Lemma 6 to *Q* with *D* replaced by *DD* ^*π*^; we have

(52) $$\begin{array}{c} Q^{d}=\left[\begin{array}{cc} A^{d} & \Gamma^{\prime }\\ \Delta^{\prime } & \Delta^{\prime }A\Gamma^{\prime } \end{array}\right], \end{array}$$

where

(53) $$\begin{array}{c} \Gamma^{\prime }=\overset{\infty }{\underset{n=0}{\sum }}{\left(A^{d}\right)}^{n+2}BD^{n}D^{\pi },\;\;\Delta^{\prime }=\overset{\infty }{\underset{n=0}{\sum }}D^{n}D^{\pi }C{\left(A^{d}\right)}^{n+2}. \end{array}$$

Observe that (50) and (51) yield

(54) $$\begin{array}{c} \Gamma =\overset{\infty }{\underset{n=0}{\sum }}{\left(A^{d}\right)}^{n+2}BD^{n},\;\;\Delta =\overset{\infty }{\underset{n=0}{\sum }}D^{n}C{\left(A^{d}\right)}^{n+2}, \end{array}$$

so we get

(55) $$\begin{array}{c} Q^{d}=\left[\begin{array}{cc} A^{d} & \Gamma \\ \Delta & \Delta A\Gamma \end{array}\right]. \end{array}$$

The condition *BD* ^*n*^ *C* = 0 implies that

(56) $$\begin{array}{c} B\Delta =B\overset{\infty }{\underset{n=0}{\sum }}D^{n}C{\left(A^{d}\right)}^{n+2}=0. \end{array}$$

Hence we have

(57) QQd=AAd+BΔA2AdΓ+BΔAΓCAd+DDπΔCΓ+DDπΔAΓ=AAdAΓCAd+DΔCΓ+DΔAΓ,Qπ=Aπ−AΓ−CAd−DΔI−CΓ−DΔAΓ.

From *AA* ^*π*^ *B* = *BD* ^2^ *D* ^*d*^ and *D* ^2^ *D* ^*d*^ *C* = *D* ^*π*^ *CAA* ^*π*^, we obtain *PQ* = *P* ^*π*^ *QPQ* ^*π*^. Applying Theorem 4, we get

(58) P+Qd=QπPd+QdPπ +∑n=0∞Qdn+2PP+QnPπ +Qπ∑n=0∞P+QnQPdn+2 −∑n=0∞ ∑k=0∞Qdk+1PP+Qn+kQPdn+2 −∑n=0∞Qdn+2PP+QnQPd,

where

(59) $$\begin{array}{c} \Gamma D^{2}D^{d}=\overset{\infty }{\underset{n=0}{\sum }}{\left(A^{d}\right)}^{n+2}BD^{n}D^{\pi }D^{2}D^{d}=0,\\ \Delta AA^{\pi }=\overset{\infty }{\underset{n=0}{\sum }}D^{n}D^{\pi }C{\left(A^{d}\right)}^{n+2}AA^{\pi }=0, \end{array}$$

so we get

(60) QdP=AdΓΔΔAΓAAπ00D2Dd=AdAAπΓD2DdΔAAπΔAΓD2Dd=0000.

Hence from (58) and (60) we obtain

(61) P+Qd=QπPd+QdPπ+Qπ∑n=0∞P+QnQPdn+2.

By direct computation we verify that

(62) $$\begin{array}{c} Q^{\pi }P^{d}=P^{d},\;\;Q^{d}P^{\pi }=Q^{d}. \end{array}$$

From *BD* ^*n*^ *C* = 0, we have

(63) ∑n=0∞P+QnQPdn+2  =0∑n=0∞AnBDdn+20∑n=1∞ ∑i=0n−1DiCAn−i−1BDdn+2.

Observe that (51) and *BD* ^*n*^ *C* = 0 yield

(64) Qπ∑n=0∞P+QnQPdn+2  =Aπ−AΓ−CAd−DΔI−CΓ−DΔAΓ0∑n=0∞AnBDdn+20∑n=1∞∑i=0n−1DiCAn−i−1BDdn+2  =0Aπ∑n=0∞AnBDdn+2−AΓ∑n=1∞∑i=0n−1DiCAn−i−1BDdn+20−CAd−DΔ∑n=0∞AnBDdn+2+I−CΓ−DΔAΓ∑n=1∞∑i=0n−1DiCAn−i−1BDdn+2  =0∑n=0∞AnBDdn+20∑n=1∞∑i=0n−1DiCAn−i−1BDdn+2.

From (62) and (64) it follows (48).

The proof is finished.

**Theorem 12 .**

Let *A* and *D* be generalized Drazin invertible and let *M* be matrix of form (26). If *AA* ^*π*^ *BD* ^*π*^ = *BD*, *BC* = 0, *CA* ^*d*^ = 0, and *CBD* ^*π*^ = 0. Then

(65) $$\begin{array}{c} M^{d}=\left[\begin{array}{cc} A^{d} & {\left(A^{d}\right)}^{2}B\\ X_{0} & D^{d}+\overset{\infty }{\underset{n=0}{\sum }}X_{n+1}BD^{n} \end{array}\right], \end{array}$$

where

(66) $$\begin{array}{c} X_{n}=\overset{\infty }{\underset{i=0}{\sum }}{\left(D^{d}\right)}^{i+n+2}CA^{i},\;n\geq 0. \end{array}$$

**Proof**

We can split matrix *M* as *M* = *P* + *Q*, where

(67) $$\begin{array}{c} P=\left[\begin{array}{cc} A^{2}A^{d} & B\\ 0 & 0 \end{array}\right],\;\;Q=\left[\begin{array}{cc} AA^{\pi } & 0\\ C & D \end{array}\right],\\ P^{d}=\left[\begin{array}{cc} A^{d} & {\left(A^{d}\right)}^{2}B\\ 0 & 0 \end{array}\right],\;\;P^{\pi }=\left[\begin{array}{cc} A^{\pi } & -A^{d}B\\ 0 & I \end{array}\right]. \end{array}$$

Applying Lemma 7, we have (*AA* ^*π*^)^*d*^ = 0, so we get

(68) $$\begin{array}{c} {\left(Q^{d}\right)}^{n}=\left[\begin{array}{cc} 0 & 0\\ X_{n-1} & {\left(D^{d}\right)}^{n} \end{array}\right],\;\;Q^{\pi }=\left[\begin{array}{cc} I & 0\\ -DX_{0} & D^{\pi } \end{array}\right], \end{array}$$

where *X*_*n*_ is defined in (28).

Since *AA* ^*π*^ *BD* ^*π*^ = *BD*, *BC* = 0, *CBD* ^*π*^ = 0, and *CA* ^2^ *A* ^*d*^ = 0, we obtain *PQ* = *P* ^*π*^ *QPQ* ^*π*^. Applying Theorem 4, we get

(69) P+Qd=QπPd+QdPπ +∑n=0∞Qdn+2PP+QnPπ +Qπ∑n=0∞P+QnQPdn+2 −∑n=0∞ ∑k=0∞Qdk+1PP+Qn+kQPdn+2 −∑n=0∞Qdn+2PP+QnQPd.

From *CA* ^*d*^ = 0, we have *QP* ^*d*^ = 0. Hence from (69) we obtain

(70) $$\begin{array}{c} {\left(P+Q\right)}^{d}=Q^{\pi }P^{d}+Q^{d}P^{\pi }+\overset{\infty }{\underset{n=0}{\sum }}{\left(Q^{d}\right)}^{n+2}P{\left(P+Q\right)}^{n}P^{\pi }, \end{array}$$

where *X*_*n*_ *A* ^*d*^ = 0, we get

(71) QπPd=Pd,  QdPπ=Qd,Qd2PPπ=00X1Dd2A2AdB00Aπ−AdB0I=000X1B.

The conditions *AA* ^*π*^ *BD* ^*π*^ = *BD* and *BC* = 0  imply that *BD* ^*i*^ *C* = 0. So we get

(72) ∑n=0∞Qdn+2PP+QnPπ =000∑n=1∞Xn+1BDn, n≥1.

From (71) and (72) it follows (65).

The proof is finished.

Using (40) and Theorem 12, we have the following result.

**Theorem 13 .**

If *CA* = *D* ^*π*^ *DCA* ^*π*^, *BD* ^*d*^ = 0, *CB* = 0, and *BCA* ^*π*^ = 0. Then

(73) $$\begin{array}{c} M^{d}=\left[\begin{array}{cc} A^{d}+\overset{\infty }{\underset{n=0}{\sum }}X_{n+2}CA^{n} & X_{1}\\ {\left(D^{d}\right)}^{2}C & D^{d} \end{array}\right], \end{array}$$

where

(74) $$\begin{array}{c} X_{n}=\overset{\infty }{\underset{i=0}{\sum }}{\left(A^{d}\right)}^{i+n+1}BD^{i},\;n\geq 1. \end{array}$$

Using the case of Theorem 3, we get the following results.

**Theorem 14 .**

Let *A* and *D* be generalized Drazin invertible and let *M* be matrix of form (26). If *DCA* ^*π*^ = *CA*, *ABD* = 0, *BC* = 0, and *CB* = 0. Then

(75) $$\begin{array}{c} M^{d}=\left[\begin{array}{cc} A^{d}+\overset{\infty }{\underset{n=0}{\sum }}B{\left(D^{d}\right)}^{n+3}CA^{n} & {\left(A^{d}\right)}^{2}B+B{\left(D^{d}\right)}^{2}\\ \overset{\infty }{\underset{n=0}{\sum }}{\left(D^{d}\right)}^{n+2}CA^{n} & D^{d} \end{array}\right]. \end{array}$$

**Proof**

We can split matrix *M* as *M* = *P* + *Q*, where

(76) $$\begin{array}{c} P=\left[\begin{array}{cc} 0 & 0\\ C & 0 \end{array}\right],\;\;Q=\left[\begin{array}{cc} A & B\\ 0 & D \end{array}\right]. \end{array}$$

From *ABD* = 0, we have

(77) $$\begin{array}{c} {\left(Q^{d}\right)}^{i}=\left[\begin{array}{cc} {\left(A^{d}\right)}^{i} & X_{i}\\ 0 & {\left(D^{d}\right)}^{i} \end{array}\right],\\ Q^{\pi }=\left[\begin{array}{cc} A^{\pi } & -AX_{1}-BD^{d}\\ 0 & D^{\pi } \end{array}\right], \end{array}$$

where

(78) $$\begin{array}{c} X_{n}={\left(A^{d}\right)}^{n+2}B+B{\left(D^{d}\right)}^{n+2},\;n\geq 0. \end{array}$$

Note that *P* is quasinilpotent; since *DCA* ^*π*^ = *CA*, *ABD* = 0, *BC* = 0, and *CB* = 0, we obtain *PQ* = *QPQ* ^*π*^. Applying Theorem 3, we get

(79) $$\begin{array}{c} {\left(P+Q\right)}^{d}=Q^{d}+\overset{\infty }{\underset{n=0}{\sum }}{\left(Q^{d}\right)}^{n+2}P{\left(P+Q\right)}^{n}. \end{array}$$

From *BC* = 0, we have

(80) Qd2P=Ad2X20Dd200C0=X2C0Dd2C0=BDd30Dd2C0.

The conditions *DCA* ^*π*^ = *CA* and *CB* = 0  imply that *CA* ^*i*^ *B* = 0. From *ABD* = 0, *CA* ^*i*^ *B* = 0, and *BC* = 0, we get

(81) ∑n=1∞Qdn+2PP+Qn =∑n=1∞Xn+2CAn∑n=1∞Xn+2CAn−1B∑n=1∞Ddn+2CAn∑n=1∞Ddn+2CAn−1B =∑n=1∞BDdn+3CAn0∑n=1∞Ddn+2CAn0.

From (77), (80), and (81) it follows (75).

Using (40) and Theorem 14, we have the following result.

**Theorem 15 .**

Let *A* and *D* be generalized Drazin invertible and let *M* be matrix of form (26). If *ABD* ^*π*^ = *BD*, *DCA* = 0, *BC* = *ABCA* ^*d*^, and *CB* = 0. Then

(82) $$\begin{array}{c} M^{d}=\left[\begin{array}{cc} A^{d} & \overset{\infty }{\underset{n=0}{\sum }}{\left(A^{d}\right)}^{n+2}BD^{n}\\ {\left(D^{d}\right)}^{2}C+C{\left(A^{d}\right)}^{2} & D^{d}+\overset{\infty }{\underset{n=0}{\sum }}C{\left(A^{d}\right)}^{n+3}BD^{n} \end{array}\right]. \end{array}$$
